# 3D-Printed Microwell Arrays for *Ciona* Microinjection and Timelapse Imaging

**DOI:** 10.1371/journal.pone.0082307

**Published:** 2013-12-06

**Authors:** Clint Gregory, Michael Veeman

**Affiliations:** Division of Biology, Kansas State University, Manhattan, Kansas, United States of America; University of North Carolina at Chapel Hill, United States of America

## Abstract

Ascidians such as *Ciona* are close chordate relatives of the vertebrates with small, simple embryonic body plans and small, simple genomes. The tractable size of the embryo offers considerable advantages for in toto imaging and quantitative analysis of morphogenesis. For functional studies, *Ciona* eggs are considerably more challenging to microinject than the much larger eggs of other model organisms such as zebrafish and *Xenopus*. One of the key difficulties is in restraining the eggs so that the microinjection needle can be easily introduced and withdrawn. Here we develop and test a device to cast wells in agarose that are each sized to hold a single egg. This injection mold is fabricated by micro-resolution stereolithography with a grid of egg-sized posts that cast corresponding wells in agarose. This 3D printing technology allows the rapid and inexpensive testing of iteratively refined prototypes. In addition to their utility in microinjection, these grids of embryo-sized wells are also valuable for timelapse imaging of multiple embryos.

## Introduction

**Figure 1:**
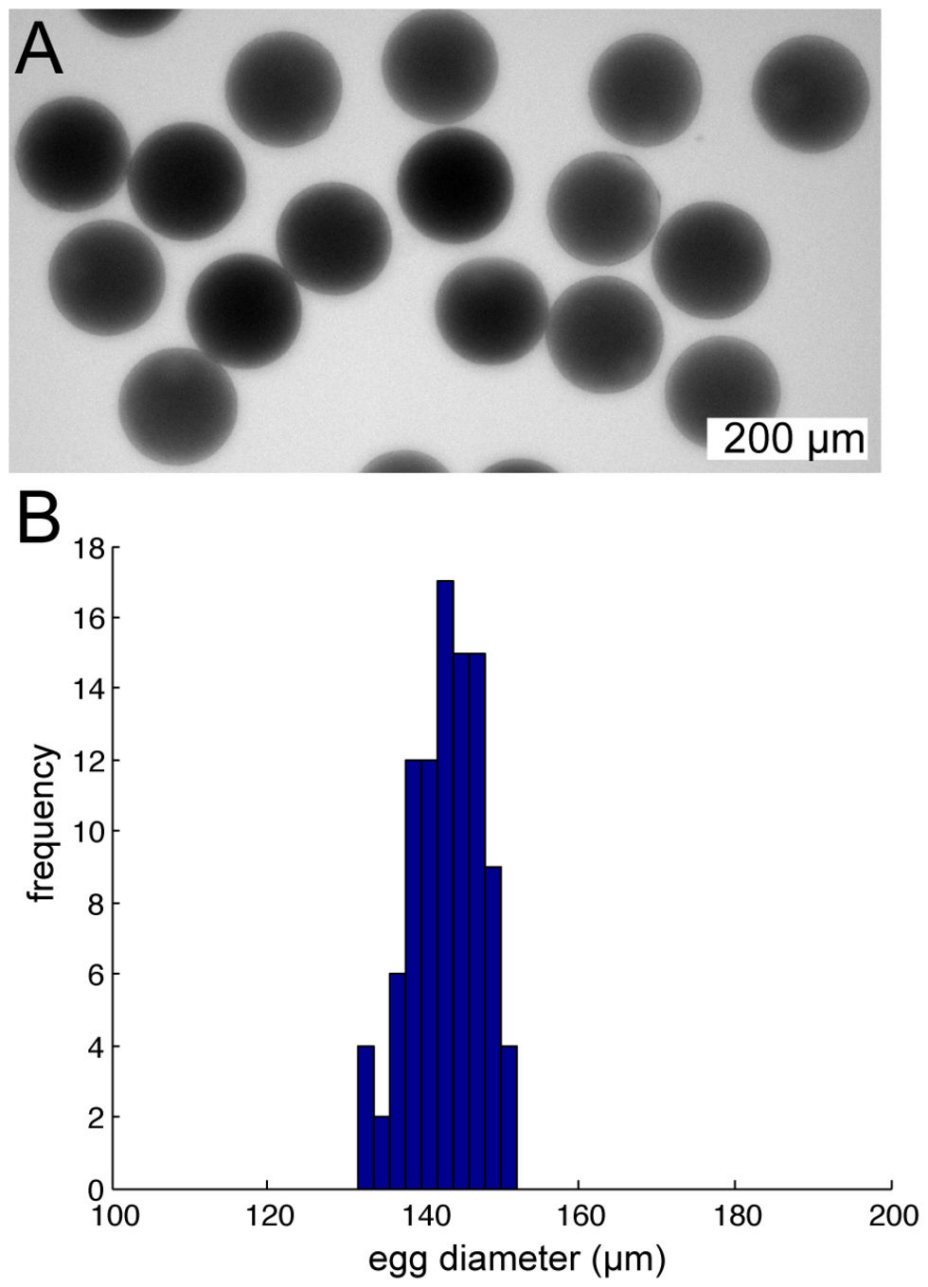
*Ciona* egg diameter. **A**) Brightfield microscope image of fertilized *Ciona intestinalis* eggs. **B**) Histogram of egg diameter for 97 measured eggs.

The *Ciona* egg is approximately 140 µm in diameter (Figure 1) and develops into an embryo with a stereotyped chordate body plan, including a notochord and hollow dorsal neural tube, that is small enough to be imaged in toto with fine subcellular detail [1]. While this small and simple embryonic body plan is advantageous for quantitative studies of embryonic morphogenesis [2–5], eggs of this size are more challenging to inject with morpholinos, RNAs and other reagents than the larger eggs of zebrafish, *Xenopus*, *Drosophila*, etc. *Ciona savignyi* eggs are surrounded by a chorion that is soft enough to be pierced by the microinjection needle and can be held in place with a holding pipette [6]. In the more commonly studied species *Ciona intestinalis* the chorion is extremely tough and needs to be chemically removed before microinjection. The dechorionated *Ciona* egg is too easily deformed to be readily held with a holding pipette. It is also too delicate to be adhered directly to the surface of a dish as is sometimes done with sea urchin eggs [7].

Dechorionated ascidian eggs can be injected horizontally after being loaded into a Kiehart wedge chamber [7,8], but this requires an unusual configuration of the microscope and micromanipulator. Another common method is to use a system of plastic blocks and coverslip fragments to cast a groove in agarose the width of the coverslip [8]. Both of these methods are fussy and require considerable skill, especially for the injection of more than a modest number of eggs.

We felt that modern microfabrication methods might allow the development of an improved device for holding ascidian eggs in place for microinjection. One such device would be an array of posts used to cast agarose wells, each well sized to hold a single egg in place. Diverse microfabrication methods have been used to build microwell arrays for trapping single organelles [9], cells [10–16], and embryos [17–19]. Several technologies exist that would allow the fabrication of arrays of posts in the 100-200 µm size range [20]. Standard microfluidic methods using PDMS soft lithography can easily create features of this size, but require access to a cleanroom with specialized equipment[21]. Various laser ablation technologies can also be used to mill features in this size range, but this again depends on expensive equipment that is not widely available [22,23]. As developmental biologists with no prior experience in microfabrication, we hoped to find a technology that was commercially available at modest cost. One promising method was to have the injection mold 3D printed from a CAD file of our own design.

There are several different 3D printing technologies that allow parts to be manufactured additively rather than subtractively as in a traditional milling or ablation operation [24]. These include methods where a melted thermoplastic is extruded from a nozzle in layers, methods where a granular material is selectively fused by heat from a laser, and methods where a laser is used to selectively polymerize a resin. At present, the best technology for printing finely-featured parts involves the photo-polymerization method, typically referred to as stereolithography.

## Results

### Design of the Injection Mold, Prototype 1

**Figure 2:**
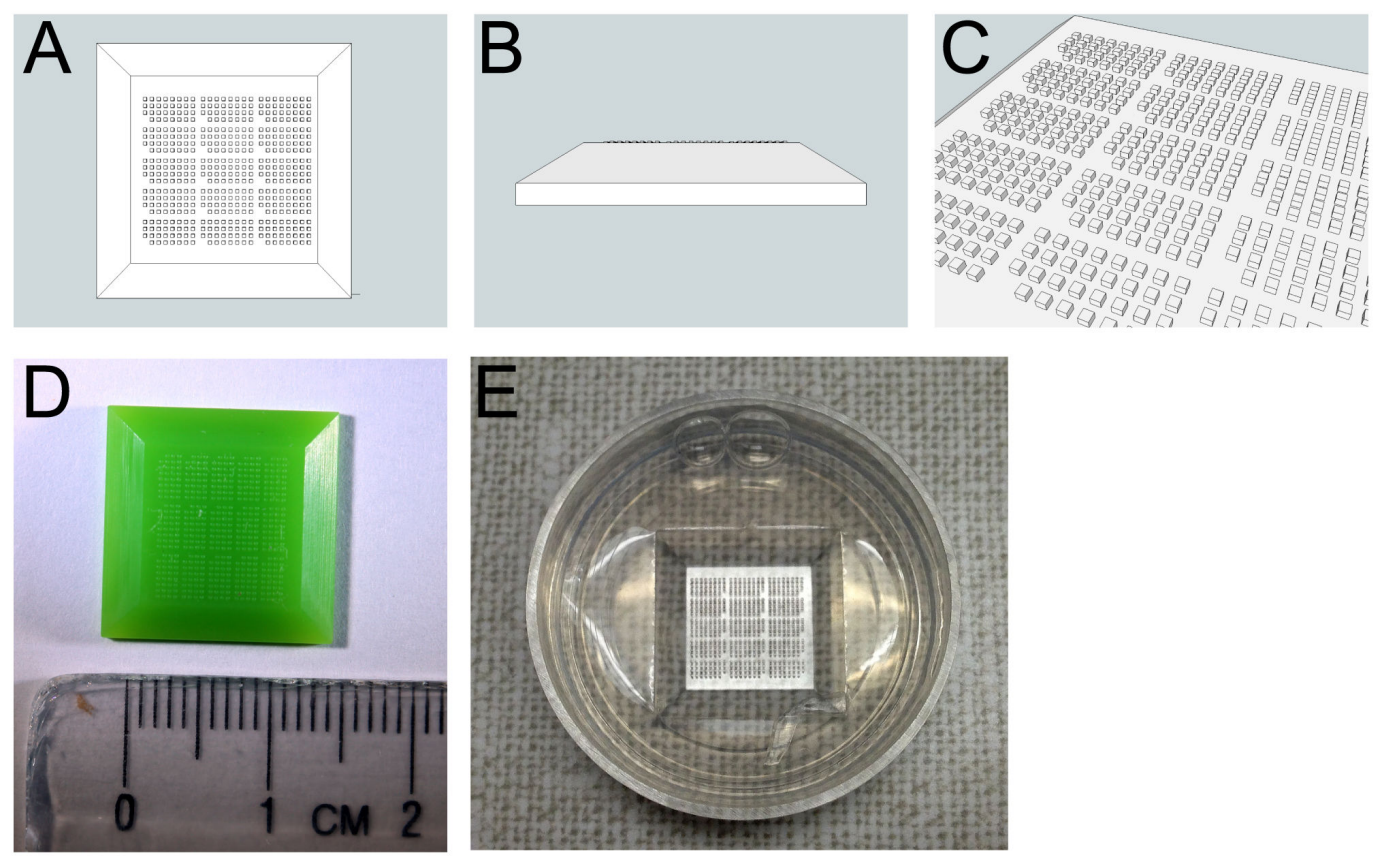
*Ciona* injection mold, prototype 1. **A**) Top view of the design for the proposed device. **B**) Side view. **C**) Perspective view. **D**) Photograph of the device as built by micro-resolution stereolithography. **E**) Agarose dish with egg-sized microwells cast using the prototype mold device.

We identified a company (Fineline Prototyping, Raleigh, North Carolina, USA) specializing in finely detailed stereolithography. Their micro-resolution method can print up to a 1" (25.4mm) cube with a minimum feature size of 0.0016" (40µm) using a proprietary material similar to ABS plastic. We designed our first generation injection mold as a thin slab sized to float on top of a layer of molten agarose in a 35mm petri dish. One side of the slab is decorated with a grid of rectangular posts (Figure 2A,B,C). For our first prototype we felt that it would be better for the posts to be slightly too large rather than too small, so we made the posts 200 µm in X and Y, and 150 µm in Z. We divided the grid into several subarrays and omitted some posts to provide indexing marks. This was done so that it would be easier to navigate around the array under the microscope, such as to revisit the same egg or embryo at multiple times. There are 12 subarrays each containing 31 posts, for a total of 372 wells in the injection dish cast from this mold. We had three of these printed for a total cost of ~$200.

### Testing Prototype 1

The injection mold printed successfully (Figure 2D) and proved easy to float on top of a layer of melted agarose to cast the array of wells (Figure 2E). In trial injection experiments, however, we identified several defects with the design. The first was that we found it difficult to "swirl' the eggs into the center of the dish so that they would fall into the wells. This was because the square shape of the mold gave rise to sharp corners that deflected the eggs in unpredictable ways when the dish was gently swirled. We also found that the 200 µm wells were considerably too large. Two eggs would sometimes jam into a single well, and the microinjection pipette had difficulty in clearing the side of the well. The eggs were also too free to roll around inside each well. We found that the taper on the sides of the square mold was too abrupt, giving rise to pipette clearance issues over much of the array. Lastly, we felt that the wells were spaced too far apart so that there were fewer eggs than desirable in any field of view with our injection microscope. While not optimal for microinjection, the larger wells in this design were advantageous for timelapse imaging as will be discussed later.

### Design of the injection mold, Prototype 2

**Figure 3:**
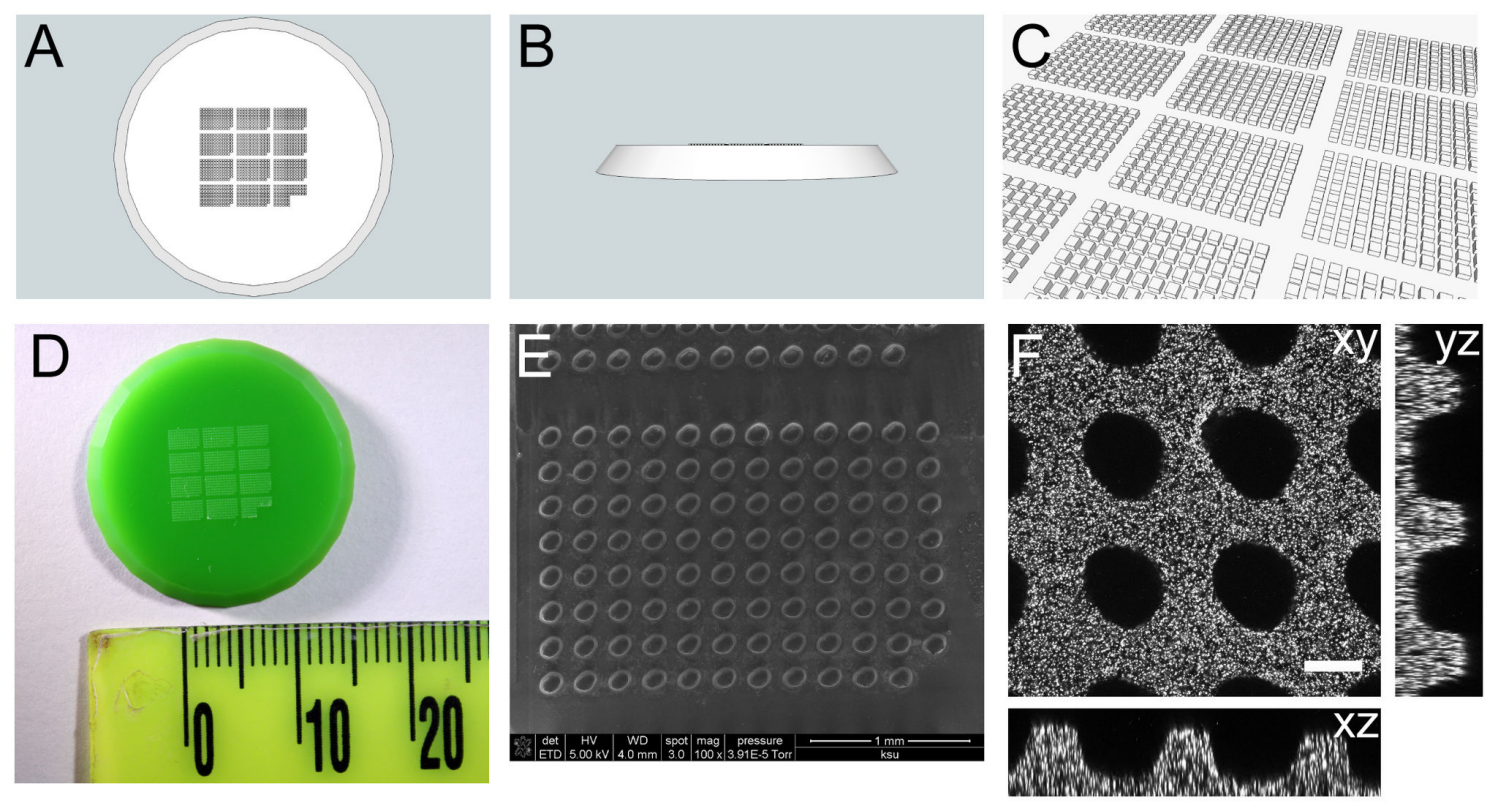
*Ciona* injection mold, prototype 2. **A**) Top view of the design for the proposed device. **B**) Side view. **C**) Perspective view. **D**) Photograph of the device as built by micro-resolution stereolithography. **E**) Scanning electron micrograph of one subarray of posts on the 3D printed device. **F**) Confocal micrograph of micro-wells cast in agarose. The agarose is labelled with fluorescent beads. Orthogonal reconstructions through the confocal stack are as indicated. Scale bar=100µm.

Our experiences with the prototype suggested some obvious improvements (Figure 3A,B,C). We redesigned the main shape of the mold to be round instead of square, with a much shallower taper on the sides. We reduced the size of the posts to be 150µm in X and Y and only 100µm in Z. We reduced the spacing between posts to 75 µm. The redesigned array still has 12 subarrays, but each of these is designed around a 96 well grid instead of a 32 well grid. Once certain posts were omitted to provide indexing marks, the final array had 1117 positions. A rendered movie illustrating the design is available as Movie S1.

### Testing Prototype 2

The revised injection mold also printed well (Figure 3D), though we noted that the printed posts came out considerably more rounded than the design file. This is likely because these smaller posts are close to the useful resolution of the printer. To better characterize the size and shape of the posts, we imaged the part by scanning electron microscopy (Figure 3E). We also performed confocal imaging of the wells as cast in agarose labeled with fluorescent beads (Figure 3F). Both methods indicated that the wells should be a good fit for gently holding *Ciona* eggs.

**Figure 4:**
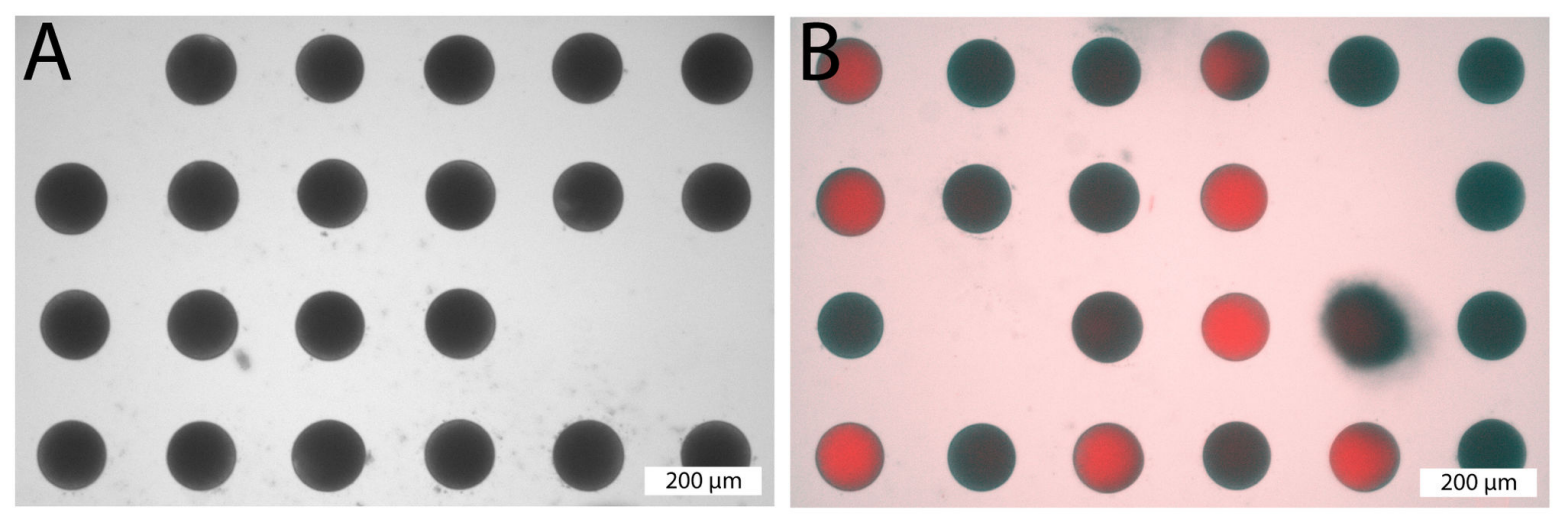
Eggs arrayed for microinjection. **A**) Uninjected eggs in the microwell array (prototype 2). **B**) Eggs injected with Alexa 568 conjugated dextran are evident by their red color (fluorescent overlay over bright field image).

We found this revised design to be extremely successful for ascidian egg microinjection (Figure 4A,B). The disk shape and gently tapered sides of the mold make it easy to swirl the embryos into the center of the dish. The resulting wells are sized correctly to snugly hold a single egg. We found it difficult to load eggs into every well of the array, but with gentle swirling and pipetting we were consistently able to load hundreds of eggs into wells. Eggs stay in the wells when undisturbed but can be easily removed by pipetting. The height of the egg inside each well was such that a microinjection needle could easily be introduced into the egg without it hitting the side of the well, and could be removed without dragging the egg from the well. Overall, we found it much easier and faster to microinject eggs held in these microwells than previous methods we had used casting fine trenches with jury-rigged devices. Movie S2 shows arrayed *Ciona* eggs being injected.

The only difficulty we had with the microwell injection trays was when we tried to let fertilized eggs develop inside the wells. *Ciona* embryos transiently become quite flat and broad at the 4 cell stage, and we found that they became somewhat deformed inside the wells at this point and subsequently developed abnormally (not shown). This could likely be avoided by making the wells slightly wider at the cost of having the eggs roll around more while being injected, or by casting the array in a more compliant gel of lower concentration low melting point agarose. For our typical uses, we find it convenient to inject unfertilized eggs in the microwell array and then remove them to be fertilized, washed and cultured in agarose-coated dishes.

### Timelapse imaging

**Figure 5:**
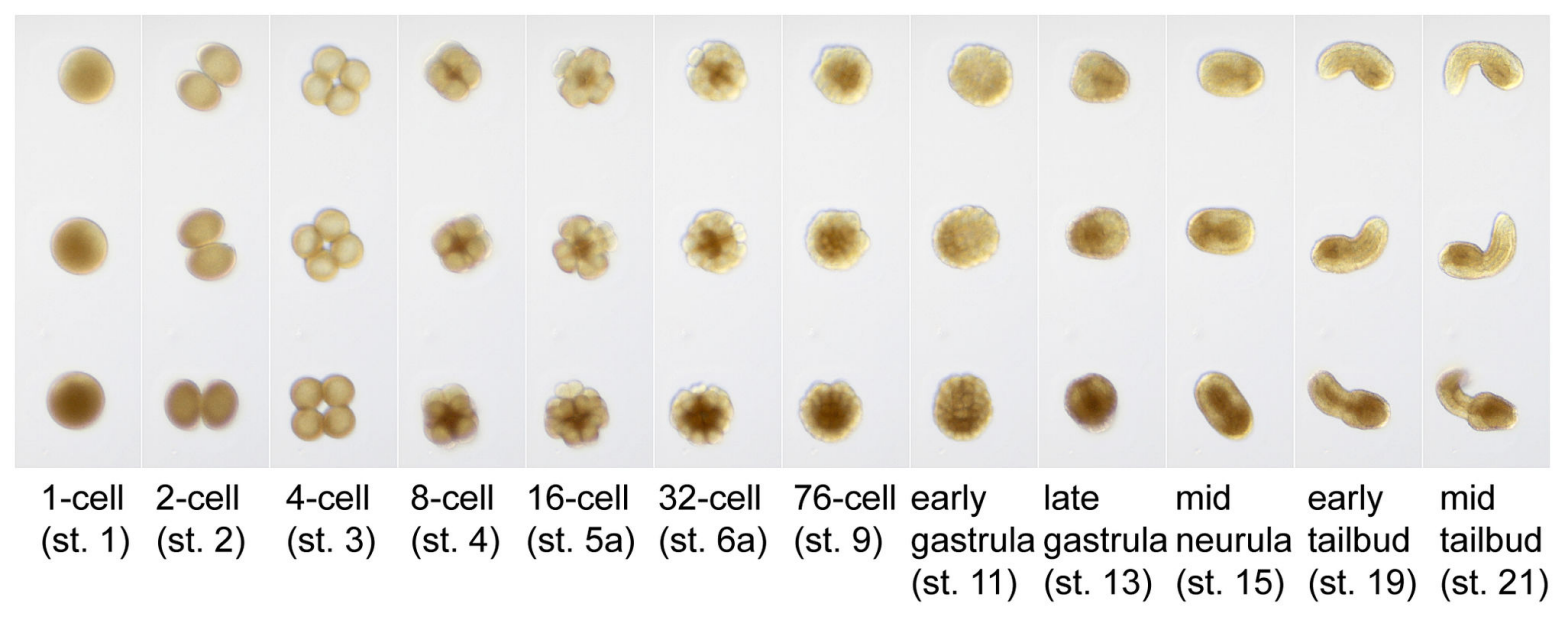
Embryos arrayed for timelapse imaging. Selected frames from a differential interference contrast time-lapse movie of fertilized eggs developing through to the mid-tailbud stage while arrayed in agarose microwells (prototype 1).

Another potential use of carefully sized microwell arrays is in timelapse imaging of embryonic development. Embryos floating loose in a dish or on a slide have a distressing tendency to drift out of the field of view at key moments. We tested the utility of our microwell arrays to restrain developing embryos for time-lapse imaging. For these experiments we used the slightly larger (200 µm) wells from our first prototype so as not to physically constrain the developing embryos. We found that embryos develop normally inside the wells but stay in one place so that they can be imaged over long periods of time. Figure 5 shows excerpts from a timelapse sequence of several embryos developing in the array between the fertilized egg and mid-tailbud stages. A movie of this sequence is available as Movie S3.

## Discussion

The embryology community has a long history of using simple homemade or custom-machined devices to handle, manipulate and perturb developing embryos. 3D printing provides a powerful new method for building custom microwell arrays to hold tiny eggs and embryos. Conventional machining operations would restrict the minimum spacing of the post array to the size of the smallest available end mill. SU-8/PDMS soft lithography, as is widely used for microfluidics, could be used to build a comparable array, but depends on having access to a cleanroom and specialized equipment. The soft lithography methods are capable of finer features, but 3D printing has sufficient resolution for the task at hand and has the advantage of being able to easily build parts that ramp or taper in the z axis.

Perhaps the biggest advantage of 3D printing in this context is that it offers an extremely straightforward and rapid design cycle. We designed our molds in Sketchup (Trimble Inc., Sunnyvale, CA), which is free and easy to learn for scientists with no previous computer aided design experience. The 3D printed parts can then be ordered over the internet at modest expense and received in just a few days. This makes it very easy to iteratively improve a design or test different possible configurations. It would be straightforward to alter the design to suit eggs of varying diameters from different species, or to change the depth, spacing, or other characteristics of the microwell array. For timelapse imaging, one could easily design embryo “corrals” sized to match the field of view of particular microscope objectives.

### Design Files

The 3D models for our two injection mold designs are available as supplemental information for this paper (combined zip archive File S1). We include the files in both sketchup format, which is more easily modified, and .stl format, which is accepted by a broader range of printing services. 

## Methods

### Designing, printing and casting the microwell array injection trays

The 3D models for the injection molds were designed in Sketchup. The parts were printed in micro-resolution mode by Fineline Prototyping using MicroFine Green resin. To cast a microwell array injection tray, we half-fill a 35 mm coverslip bottomed dish with melted 1% agarose in artificial seawater and then float the injection mold on top, with the array of posts facing downwards. The coverslip bottomed dish is not essential but gives better transmitted light images. When the agarose has solidified, the mold can be gently pried off with forceps. The plastic mold should then be rinsed with distilled water and left to dry. We have not noticed any obvious wear or deterioration on the molds over many uses. The agarose dish with the microwell array should be immediately filled with seawater so that the wells do not dry out and distort.

### Characterizing the arrays

Scanning electron microscopy of the 3D printed device was carried out on a FEI Nova NanoSEM 430. Confocal microscopy of the agarose microwells was carried out on a Zeiss LSM 700 using a 20x 0.5NA air objective. The agarose was labeled with 0.2µm yellow-green fluorescent microspheres (Invitrogen).

### Loading the microwell array

Dechorionated eggs can be pipetted into the microwell injection dishes using a 200 µl pipettor with a tip that has been cut back and coated with 0.1% BSA. Gentle swirling will move the eggs into the center of the dish where they will fall into the wells. This can be enhanced by gently moving the eggs around in the center of the dish using a pipettor held at an oblique angle.

### Injections

Injections were performed using a custom micromanipulator and an Olympus BX51wi upright nosepiece focusing microscope. The manipulator uses three micrometer-driven Thorlabs translation stages for the X, Y and Z axes and a motor-driven stage for the approach axis. Needles were pulled on a Sutter P-97 using 1.0mm thinwalled tubing with a loading filament. The needles were backloaded with Alexa 568 dextran dye solution and the tips were gently broken back on a small (~2mmx2mm) fragment of microscope slide placed in the injection dish. Pneumatic pressure injections used a Harvard Apparatus PLI-100 microinjector. This model of microinjector has a vacuum feature that makes it easy to apply the brief pulse of negative pressure that *Ciona* eggs require in order to break the egg membrane. 

### Timelapse Imaging

Fertilized and dechorionated *Ciona* eggs were loaded into the array and imaged on an Olympus BX51wi microscope using a 10x 0.3NA objective and differential interference contrast optics. Images were collected every 15 seconds using a Canon T3i camera controlled by DSLR Remote Pro software (Breeze Systems). Imaging from the top of an open dish, we found that our timelapses were ultimately limited by the salt concentration rising as water evaporated from the dish. This could possibly be improved with an oil overlay or perfusion system for >12hour timelapses.

## Supporting Information

File S1 **Zip archive of design files.**
(ZIP)

Movie S1 **3D rendering of prototype 2.** This movie provides 3D rendered views of the design for our second prototype injection mold.
(MOV)

Movie S2 **Microinjection of arrayed *Ciona* eggs.** This movie shows eggs being loaded into the microwell array and then microinjected with a fluorescent dye.
(MOV)

Movie S3 **Developmental timelapse of arrayed *Ciona* embryos.** This movie shows embryos developing in the array from fertilized egg through mid-tailbud stages.
(MOV)
